# Tumor Microenvironment-Based Stimuli-Responsive Nanoparticles for Controlled Release of Drugs in Cancer Therapy

**DOI:** 10.3390/pharmaceutics14112346

**Published:** 2022-10-31

**Authors:** Weixin Zhou, Yujie Jia, Yani Liu, Yan Chen, Pengxuan Zhao

**Affiliations:** 1 Department of Medical Ultrasound, Tongji Hospital, Tongji Medical College, Huazhong University of Science and Technology, Wuhan 430030, China; 2 School of Pharmacy, Tongji Medical College, Huazhong University of Science and Technology, Wuhan 430030, China; 3 Institute of Biomedical Engineering and Technology, Shanghai Engineering Research Center of Molecular Therapeutics and New Drug Development, School of Chemistry and Molecular Engineering, East China Normal University, Shanghai 200065, China

**Keywords:** cancer therapy, tumor microenvironment, stimuli-responsive, nanoparticles

## Abstract

With the development of nanomedicine technology, stimuli-responsive nanocarriers play an increasingly important role in antitumor therapy. Compared with the normal physiological environment, the tumor microenvironment (TME) possesses several unique properties, including acidity, high glutathione (GSH) concentration, hypoxia, over-expressed enzymes and excessive reactive oxygen species (ROS), which are closely related to the occurrence and development of tumors. However, on the other hand, these properties could also be harnessed for smart drug delivery systems to release drugs specifically in tumor tissues. Stimuli-responsive nanoparticles (srNPs) can maintain stability at physiological conditions, while they could be triggered rapidly to release drugs by specific stimuli to prolong blood circulation and enhance cancer cellular uptake, thus achieving excellent therapeutic performance and improved biosafety. This review focuses on the design of srNPs based on several stimuli in the TME for the delivery of antitumor drugs. In addition, the challenges and prospects for the development of srNPs are discussed, which can possibly inspire researchers to develop srNPs for clinical applications in the future.

## 1. Introduction

Cancer, also known as malignant tumor, is still one of the common causes of human death worldwide [1]. According to the GLOBOCAN database, the number of new cancer cases was estimated to be 19.29 million, and that of cancer deaths was approximately 9.96 million, in 2020 [2]. Numerous efforts from different fields have been made to explore effective and safe strategies to treat this disease. Among various treatments, chemotherapy is one of the most commonly used methods for cancer therapy at present [3]. For the past decades, researchers have been working to deliver anti-cancer drugs to tumor sites. However, the clinical application of conventional chemotherapeutic drugs is restricted, owing to the lack of selectivity, limited water solubility, poor targeting ability and serious systemic toxicity.

**Scheme 1:**
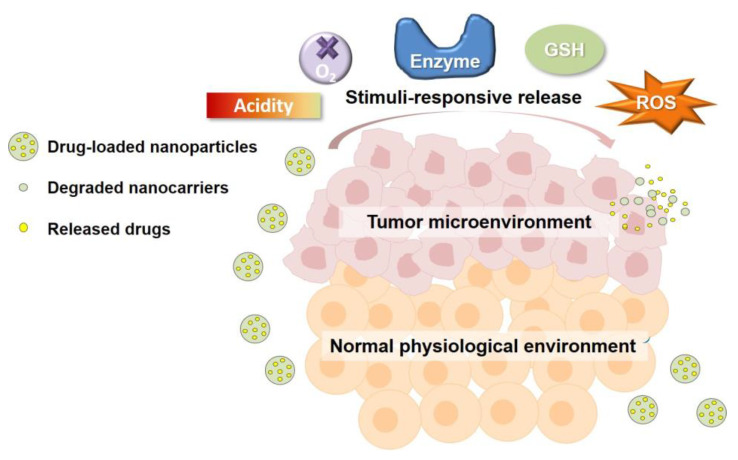
Schematic illustration of srNPs for controlled drug release in cancer treatment.

In recent years, nanomedicine has been extensively used in tumor-targeting drug delivery due to its unique molecular properties. Despite nano drug delivery systems’ (NDDS) enhancement of the efficiency of conventional chemotherapeutics, it is still an urgent problem to improve the bioavailability of drugs in tumor tissues, especially to enhance the cellular uptake and intracellular release of drugs. With a deeper understanding of the different properties between normal tissues and tumor tissues, srNPs were rationally designed for drug delivery. Thus, to better understand the advances in the srNPs for anticancer therapy, this review briefly introduced the endogenous stimuli (i.e., low pH, high GSH concentration, overexpressed enzymes, excessive ROS and hypoxia) of the TME (Scheme 1), and summarized the application of srNPs in tumor therapy, aiming to provide inspiration for further research and facilitate the clinical translation of srNPs.

## 2. Stimuli in the TME

The TME is a cellular environment composed of tumor cells, fibroblasts, lymphocytes, immune cells, bone marrow-derived inflammatory cells, signal molecules, an extracellular matrix (ECM) and surrounding blood vessels [4,5]. All the cells embedded in the ECM consists of collagen and proteoglycan. In general, the tumor vessels are characterized by irregular shape as well as loose structure, and even lack the endothelial cells or basement membrane in the malignant lesions. Therefore, the TME provides suitable conditions for tumor cells to exchange materials and promotes their proliferation and metastasis. The TME is characterized by several features, such as acidity, high GSH concentration, hypoxia, overexpressed enzymes and excessive ROS, compared to the physiological environment [6].

### 2.1. Acidity

The lower pH in the extracellular matrix and interstitial space is a sign of malignant tumor, which is caused by excessive metabolites, such as carbon dioxide, lactic acid, as well as activated vacuolar-type (V-type) H(+)-ATPases (a proton pumps) [7,8]. In general, cancer cells produce large amounts of lactic acid due to their heavy reliance on glycolysis instead of oxidative phosphorylation, and this phenomenon is called the Warburg effect [9,10,11]. Typically, the pH value in the TME (pH 6.5) is lower than that in normal cells (pH 7.4), and the abnormal pH conditions further appeared in organelles, such as nucleosome (pH 5.5) and lysosome (pH 5.0) [12]. Acidic TME has been proved to facilitate the occurrence and metastasis of tumors. In addition, the abnormal pH is also one of the causes of tumor multidrug resistance (MDR), especially for weakly alkaline chemotherapeutic drugs [13]. However, the acidity of the TME and nuclear endosome/lysosome could also be utilized as endogenous triggers for srNPs.

### 2.2. High GSH Concentration

GSH is a thiol substance composed of glutamate, glycine and cysteine. It is the most abundant reductant in living cells, especially in some organelles such as cytosol, mitochondria and the nucleus. Normal concentration of GSH, with detoxification and antioxidant effects, is crucial for the body to maintain immune system functions. The intracellular concentration of GSH is about 2–10 mM, which is significantly higher than its concentration in blood and the extracellular matrix (about 2–20 μM). In addition, tumor tissues showed 10 times higher GSH concentration than normal tissues. It has been reported that abnormal GSH levels are related to many human diseases, such as liver-related diseases, neurodegenerative diseases, epilepsy, diabetes and so on [14,15,16].

### 2.3. Hypoxia

As a hallmark of solid tumors, hypoxia is closely related to tumor invasion, metastasis and drug resistance. Due to the irregular shape of blood vessels in solid tumors, it is unable to deliver enough oxygen and nutrition to all regions, resulting in temporary or long-term hypoxia of tumor cells. The oxygen partial pressure in normal tissues is about 30 mmHg, while that in tumor tissues gradually decreases from the surface to the inside and reaches a low level (5 mmHg) in some regions, and the oxygen partial pressure in some solid tumors may be close to 0 mmHg. Moreover, Oxygen utilization also decreased with increased distance between tumor cells and blood vessels. Tumor cells in hypoxic areas divide more slowly than those in normoxic areas, making them less sensitive to chemotherapeutic drugs targeting cells that rapidly proliferate [17,18].

Moreover, hypoxia in the TME can also upregulate hypoxia-inducible factors (HIFs), protein dimerization consisting of HIF-α (oxygen-sensitive subunit) and HIF-β (constitutively expressed subunit), which can facilitate the growth and metastasis of tumors [19]. It has been reported that a HIF-α isoform stimulated tumor progression in some tumor models, such as kidney cancer and neuroblastoma. Under acute and severe hypoxia conditions (1–2% O_2_), HIF-1α could be activated promptly to combine with HIF-β [20], so the stability of HIF-1α and transcriptional activity of HIF-1 are significantly enhanced in hypoxic TME, thus increasing the expression of vascular endothelial cell growth factor (VEGF) that can promote the growth of tumors with angiogenesis [21]. In addition, increased HIF-1α can also induce immune escape [22]. Although hypoxia provides favorable conditions for tumor progression, this characteristic also provides opportunities to develop srNPs.

### 2.4. Overexpressed Specific Enzyme

Since physiological and metabolic processes in the human body depend on enzymes, the abnormal expression and activity of enzymes are the pathological basis of many diseases. Compared with that in normal tissues, some enzymes are overexpressed in tumor tissues, thus showing excessive secretion in the TME, such as matrix metalloproteinases (MMPs), hyaluronidases, β-Glucosidase, esterase [23,24]. By modification with specific enzyme substrates, srNPs can be cleaved by target enzymes and release drugs in the TME.

### 2.5. Excessive ROS

ROS include hydroxyl radicals (•OH), singlet oxygen (^1^O_2_), hydrogen peroxide (H_2_O_2_), peroxynitrite (ONOO^−^), superoxide anion (•O_2_^−^) and so on [25]. There are several endogenous sources of ROS, while they are mainly produced by the incomplete reduction of oxygen and nicotinamide adenine dinucleotide phosphate oxidase in the mitochondria and plasma membrane [26]. As a signal molecule, ROS played an important role in protein translation, transcription and survival, as well as tumorigenesis and proliferation [27]. In an appropriate concentration, ROS are the significant signal molecules for multiple metabolic pathways, while excessive ROS might damage the tissues or organs and even induce serious diseases such as cancer. As a prominent feature of cancer, hypoxia significantly changes the ROS level in tumor tissues, so the ROS concentration in tumor cells (10^−4^ M) is much higher than that in normal tissues (2 × 10^−8^ M) [28,29].

## 3. Stimuli-Responsive Nanoparticles

As mentioned above, the TME possesses a variety of unique properties and plays a critical role in the occurrence, invasion and metastasis of tumors. However, the characteristic difference between tumor tissues and normal tissues is also the theoretical basis for designing intelligent responsive NDDS [30,31]. srNPs can respond to various endogenous stimuli in the TME, and their properties, such as shape, size, surface charge and hydrophilicity, could be changed after reaching the tumor areas [32]. These changes can promote the accumulation, penetration, cellular uptake or drug release of nanoparticles, and ultimately enhance the anti-tumor therapeutic effect.

### 3.1. pH-Responsive Nanocarriers

Due to the Warburg effect, the acidic microenvironment of solid tumors can be used to achieve tumor-specific delivery of srNPs [1]. There are three common strategies for the construction of pH-responsive srNPs (Table 1). The first strategy is to use some specific molecular structures in the design of nanocarriers. The pKa values of these structures are close to the pH of the intercellular matrix, so their functional groups can be protonated in the TME with a lower pH, resulting in the destruction of the hydrophilic–hydrophobic balance of nanoparticles, thus causing structural changes, including rearrangement, expansion or disintegration [33]. Typical acid-sensitive groups include histidine, tertiary amine and sulfonamide structures. The second strategy is based on acid-labile chemical bonds, which can stably exist under neutral conditions and break under acidic conditions [34], enabling srNPs to disintegrate and release drugs in the TME. The third strategy is to use pH (low) insertion peptides (pHLIP), which could weakly interact with the cell membrane under neutral conditions and insert into the cell membrane and form stable transmembrane complexes in an acidic environment, thus promoting the cellular uptake of nanoparticles [35].

#### 3.1.1. Hydrophobic-to-Hydrophilic Transition

**Figure 1:**
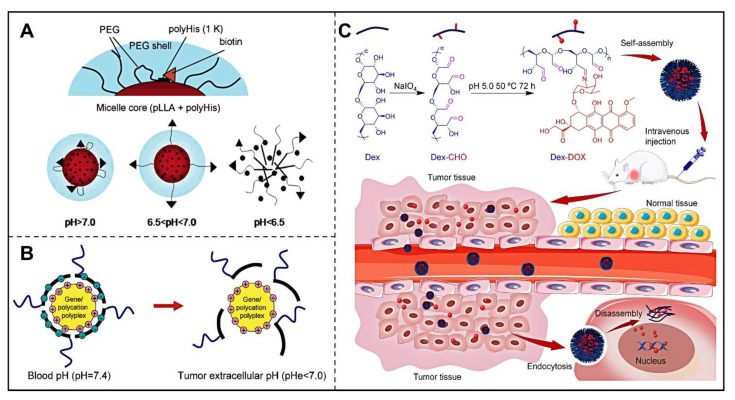
Design and responsive mechanism of pH-triggered srNPs. (**A**) Schematic depicting the pH-induced change of the micelle based on polyHis. Reproduced with permission [45]. Copyright 2005, American Chemical Society. (**B**) Illustration of the pH-responsive shielding/deshielding of polymeric nanoparticles containing sulfonamide at different pH. Reproduced with permission [51]. Copyright 2006, American Chemical Society. (**C**) Self-assembly and in vivo pH-triggered disassembly of Dex-DOX connected by the imine bond. Reproduced with permission [70]. Copyright 2017, Elsevier.

There were numerous studies on the transition from hydrophobic to hydrophilic chemical groups in the TME [36,37,38]. For example, polymers with amino groups possess this property, as the amino groups in the structure can accept a proton and become hydrophilic when the pH value of the environment drops below its pKa [39]. The typical drug based on this concept is the polyhistidine nanomicelle developed by BAE et al. [40,41,42]. Poly-L-Histidine (polyHis) is a polypeptide containing imidazole groups, of which the pKa value is 6.5, thus, it shows a reversible hydrophilic–hydrophobic transition according to its protonated and deprotonated states. Bae et al. synthesized a pH-triggered micelle by using poly(histidine-co-phenylalanine)-b-poly(ethylene glycol) and poly(L-lactic acid)-b-poly(ethylene glycol)-folate [43]. The micelles loaded drugs through the hydrophobic interaction between the anticancer drug doxorubicin (DOX) and the deprotonated polyHis fragment, so it can exist stably in physiological environments with pH 7.4. However, when the nanomicelles reach the acidic TME, the polyHis block in the core gradually becomes unstable due to protonation, thus dissociating and selectively releasing the drug. Compared with pH-insensitive micelles, these nanomicelles were able to significantly enhance the antitumor efficacy of DOX [44]. PolyHis polymers are also used for achieving pH-responsive cellular uptake (Figure 1A) [45]. These nanomicelles are not affected by protein binding under blood circulation, and they are able to expose targeting ligands and bind to overexpressed cell surface receptors after being transported to the TME, thereby enhancing the uptake of nanodrugs by cancer cells.

More recently, PolyHis-based polymers were also used in gene anti-cancer therapy [46]. Zhao et al. developed a pH-triggered nanoplatform, PHD/PLL/siRNA nanoparticle (PHD/PLL/siRNA NP), via self-assembly of methoxy poly(ethylene glycol)-polyHis-poly(sulfadimethoxine) (PHD), poly-L-lysine (PLL) and PLK1 siRNA (siPLK1) [47]. Specifically, the spontaneous formation of PHD/PLL/siRNA NP is owing to electrostatic interaction between negatively charged PHD and siPLK1 and positively charged dendritic PLL. PHD/PLL/siRNA showed higher cellular uptake and efficient endo/lysosomal escape, which can be attributed to the pH-induced protonation and proton sponge effect of the imidazole ring of polyHis. In addition, the intracellular acidic microenvironment could change the poly(sulfadimethoxine) (PSD) block from negatively charged to neutral, thus accelerating the disassembly of NPs and release of siPLK1. The results of the assay indicated that PHD/PLL/siRNA NP showed an excellent tumor elimination effect for non-small cell lung cancer therapy.

In addition to polyHis, protonation of tertiary amine groups can also alter srNPs from hydrophobicity to hydrophilicity in the acidic TME [48], thus changing the structure of nanoparticles and releasing the encapsulated drugs. For instance, Yang synthesized aliphatic polyester using enzyme polymerization [49], which could load DOX by self-assembly in aqueous solutions and release it in the TME. It was proven that the DOX release rate of micelles under an acid condition (pH 6.8) was significantly faster than that in a normal physiological environment (pH 7.4). The results of in vitro cellular uptake and cytotoxicity proved the feasibility of the drug delivery system. The pH-responsive release of drugs was owing to the transition of tertiary amine groups from hydrophobic to hydrophilic and the subsequent dissociation of structure. Zhang et al. designed a 4-diethylaminophenyl isothiocyanate (DAITC)-modified generation-five polyamidoamine (PAMAM) dendrimer (GDA) for delivering protein [50]. In this study, green fluorescent protein (EGFP) was encapsulated in GDA via complexation, and the hydrophobically modified GDA/EGFP can maintain stability at physiological pH (7.4) but disassemble at endolysosomal pH (6.0) due to the protonation of tertiary amines, thus showing high tolerability in serum and rapid release in the cytosol.

Moreover, the secondary amine group attached to the sulfone group in the sulfonamide possesses a near-neutral pKa, which can be utilized to design pH-responsive nanocarriers (Figure 1B) [51]. There exists electrostatic adsorption between negatively charged sulfonamides and positively charged polymers when pH is above 6.8, while in acidic microenvironments (pH below 6.8), the nanocarriers dissociate, as the sulfonamide is no longer charged, and the electrostatic interactions disappear. Kang and Bae designed a nanocarrier to transport and release nucleic acids using oligomeric sulfonamides (OSASs) [52], which could alter from hydrophilic to hydrophobic in a low pH environment. The nanocarriers were used to load the nucleic acid solution by formatting an OSA-polyplex. The results indicated that the OSA-polyplexes significantly enhanced DNA transfection by inducing endosomal release.

#### 3.1.2. Acid-Labile Bond Cleavage

Several nanocarriers have been conjugated with acid-labile bonds, such as hydrazones [53,54], orthoesters [55,56], imines [57,58] and acetals/ketals [59,60], so as to develop a series of pH-responsive srNPs. The hydrazone bond is one of the most commonly used acid-sensitive bonds in nano delivery systems. At pH 7.4, the hydrazone is relatively stable, whereas it rapidly hydrolyzes in endosomes and lysosomes (pH 5–6) as its C=N double bond breaks under an acidic condition [61]. Etrych is the first researcher to study hydrazone linkage [62], and prepared pH-sensitive nanomedicine exhibiting advantages such as high drug loading capacity, simple preparation process, TME-responsive drug release and enhanced antitumor activity [63]. Zhou et al. connected amphiphilic conjugates DOX and β-sitosterol to the N-(2-hydroxypropyl) methacrylamide (HPMA) polymer using hydrazone as linkage [64]. The hydrazone linkage remains quite stable throughout the blood circulation at pH 7.4, while it breaks down quickly and releases 80% of its drug at pH 5.0. The pH-responsive cross-linked micelles showed significantly enhanced tumor permeability and anti-tumor efficacy in an H22 mouse xenograft model. The results proved that HPMA polymer micelles with hydrazone connections are feasible carriers for controlled-release nanodrugs.

Liao et al. developed the pH-sensitive hyaluronic acid-hydrazone linkage-DOX NPs (HA-hyd-DOX NPs) for targeted delivery of DOX. In specific, hydrophobic DOX was conjugated with the hydrophilic HA backbone by the pH-responsive hydrazone linkage, and the HA-hyd-DOX conjugates could self-assemble into HA-hyd-DOX NPs, which can target tumors through a High Affinity of HA for overexpressed CD44 and achieve intracellular DOX release via cleavage of the hydrazone bond [65].

Not only a hydrazone bond, orthoester is also a kind of commonly used acid sensitive bond, due to its sensitivity to pH changes and its biocompatible degradation products. Huang et al. first reported polymers with orthoester as linkage [66,67]. Xu et al. synthesized a polymer poly(n-((2-(2-(dimethylamino)ethoxy)-1,3-dioxolan-4-yl)methyl)methacrylamide)(PMAOE) linked by orthoester linkages to deliver DNA [68]. Drug-loaded nanoparticles were stabilized by electrostatic interactions between cationic amine groups and anionic DNA, and they were able to promote efficient release of DNA in the TME. Nuclear magnetic resonance (NMR) assay results showed that about 60% of the side chains underwent hydrolysis at pH 4.0 and slowly released adsorbed DNA.

**Figure 2:**
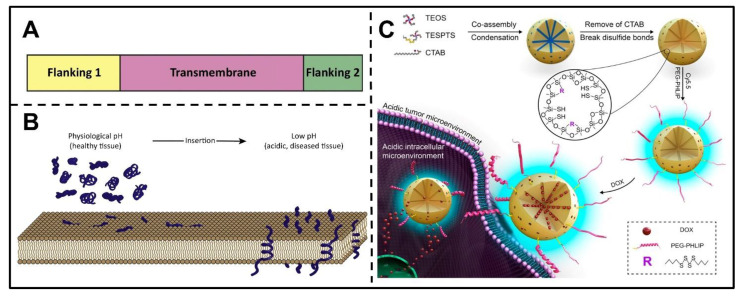
Schematic representing structure and pH-sensitive transmembrane mechanism of pHLIP. (**A**) The main features of three sequences of pHLIP. (**B**) Schematic model of membrane interaction at physiological pH and insertion at low pH of pHLIP. Reproduced with permission [83]. Copyright 2017, Elsevier. (**C**) Preparation of pHLIP-modified MONs and their targeting cancer therapy by pH-triggered transmembrane behavior. Reproduced with permission [81]. Copyright 2017, American Chemical Society.

The acid-labile imine linkage in an acidic environment has also been used as an acid-sensitive responsive bond for nano drug-loaded systems [69]. In 2017, Ding et al. fabricated dextran-doxorubicin (Dex-DOX) nanoparticles based on imine linkage (Figure 2A) [70]. Specifically, the hydroxyl groups on dextran were oxidized to aldehydes before being linked with DOX via imine linkage. The conjugate could self-assemble into uniform nanoparticles in an aqueous solution. The results proved that Dex-DOX could significantly improve cancer cellular uptake and enhance antitumor efficacy for B16-F10-bearing mice with excellent therapeutic safety. To further increase the stability of imine linkage under physiological conditions, benzoic-imide bonds were constructed by conjugation of the π–π bonds. Liao et al. developed the pH-responsive polymer nanogels with benzoic-imide cross-linkages synthesized by crosslinking of terephthalaldehyde (TPA) molecules with branched poly(ethylenimine)-g-methoxy poly(ethylene glycol) copolymers for delivering indocyanine green (ICG) [71]. The nanogel encapsulating efficiently ICG retarded leakage of drugs at pH 7.8 by improving the stability of the drug in a neutral phosphate buffer, while it released ICG with the cleavage of benzoic–imide bonds in the nanogel when the pH was changed from 7.8 to 6.4, thus achieving controlled drug release.

In addition, Gillies and colleagues first reported the use of acetals as the connecting bonds of pH-responsive srNPs in 2004 [72]. Under acidic conditions, one of the oxygen atoms of the acetal bond is protonated and induces hydrolytic fracture, resulting in the transition of nanoparticles from hydrophobic to hydrophilic, thus accelerating drug release. Recently, Wagner et al. constructed pH-triggered mesoporous silica nanoparticles (MSNs) with acetal linker for loading and controlled release of resiquimod (R848), a Toll-like receptor 7 and 8 agonist, and OVA [73]. In this research, the carboxylated surface of MSNs enables them to connect with pH-induced capping composed of an acetal linker and biotin-avidin complex. The results of tests indicated that the R848-loaded MSNs showed rapid uptake by immune cells and efficient immune activation under acidic conditions. In addition, MSN-R848-OVAp activated an enhanced specific T-cell response by codelivery of the adjuvant and antigen. To prepare the polymeric material with acid-sensitivity and biodegradability, acetylated-dextran (Ac-Dex) is obtained by replacing 73% of hydroxyl groups in dextran with acetals [74]. Attractively, at 37 °C and pH 5.0, the half-life of FITC-dextran was approximately 10 h, whereas at pH 7.4 it was approximately 15 days. At present, a variety of drugs including DOX [75], plasmid DNA [76], siRNA [77,78] and antimicrobial agents [79] have been loaded into Ac-Dex-based nanoparticles.

**Table 1:** pH-responsive srNPs in cancer therapy.

Responsive Moiety	Nanoplatform	Cargos	Application	Tumor Model	Refs.
polyHis	poly(L-lactic acid)-b-PEG-b-polyHis micelles	DOX	pH-dependent drug release	MCF-7	[40]
	polymeric micelles constitute of two block copolymers of poly(L-lactic acid)-b-PEG-b-poly(L-histidine)-TAT and polyHis-b-PEG	DOX	pH-dependent drug release and tumor targeted chemotherapy	A2780/AD, MCF-7, and A549	[42]
	A mixed-micelle system composed of polyHis-co-phenylalanine-b-poly(PEG) and poly(L-lactic acid)-b-PEG-folate	DOX	Reversal of multidrug resistance of cancer	A2780/DOXR	[43]
	a mixture of polyHis/PEG-folate and poly(L-lactic acid)-b-PEG-folate	DOX	Reversal of resistant MCF-7 tumor	MCF-7/DOXR	[44]
	A micelle composed of polyHis-b- PEG and poly(L-lactic acid)-b-PEG-b-polyHis-biotin	DOX	Increase of endocytosis.	MCF-7	[45]
tertiary amine	mPEG/HCou-g-MPCL micelles	DOX	pH-sensitive drug delivery	HeLa	[49]
	GDA/EGFP	EGFP	pH-responsive cytosolic protein delivery	143B	[50]
sulfonamide	DNA/PEI/poly(methacyloyl sulfadimethoxine)-b-PEG	DNA	Tumor specific gene delivery	A2780	[51]
	Oligomeric sulfonamides-incorporated poly(L-lysine)/DNA	DNA	enhancement of nucleic acid delivery.	HEK293	[52]
hydrazone	HPMA	DOX	pH-sensitive drug release	EL4	[62]
	HPMA	DOX β-sitosterol	pH-sensitive tumor chemotherapy	Hep G2, A549 and H22	[64]
	HA-hyd-DOX	DOX	pH-dependent drug release and tumor targeted chemotherapy	Hela	[65]
orthoester	PEG-b-PtNEA27/56/73	Nile Red.	Acid-sensitive and thermoresponsive drug release	NA	[66]
	PMAOE	DNA	pH-modulated release of gene	NA	[68]
imine	Dex-DOX	DOX	pH-sensitive tumor chemotherapy	B16F10	[70]
benzoic-imine	benzoic-imine-containing PEI-g-mPEG	ICG	Acid-triggered photoinitiation release	NA	[71]
acetals	MSN−R848−OVAp	R848 and OVA	pH-sensitive tumor immunotherapy	NA	[73]
	Ac-DEX	pyrene	pH-dependent drug release	NA	[74]
pHLIP	HauNS-pHLIP-Ce6	Ce6	Tumor targeted PTT/PDT	Hela	[80]
	MONs	DOX	Tumor targeted chemotherapy	MDA-MB-231, MCF-7	[81]

#### 3.1.3. pH(Low) Insertion Peptides

There is another typical reported approach to achieve the pH-triggered srNPs. The acidic TME can also activate pHLIPs, which are polypeptides with specific sequences that can be inserted into the cell membrane under acidic conditions [35,82]. These polypeptides are composed of two flanking sequences at the end and a transmembrane sequence in the middle (Figure 2A). The flanking sequence endows the protein with water solubility, while the transmembrane sequence is mainly composed of aspartic acid (ASP) and glutamic acid (Glu) residues, which can enable the pHLIPs to be more hydrophobic at acidic pH, thus enhancing the interaction with the cell membrane [32]. Through the mechanism of membrane-associated folding [83], pHLIPs could be triggered by the acidic TME and spontaneously form a helix to insert and span the cellular membrane (Figure 2B). Under neutral or alkaline pH conditions, pHLIPs generally exist in the form of unstructured monomers, so they can be soluble in aqueous solutions and reversibly associate with lipid bilayers or the outer surface of cell membranes. However, in the acidic microenvironment, the increased proton concentration could induce protonation of the carboxyl groups of ASP and Glu in the C-terminal and transmembrane sequences of pHLIPs, thereby enhancing their hydrophobicity. Thus, the protonation of residues triggers the formation of a transmembrane helix, which can insert itself into the hydrophobic layer of the cell membrane. The insertion is mainly directional. Usually, the C-terminal enters the cytoplasm across the bilayer membrane, while the N-terminal remains outside the cell [84,85]. It was reported that the affinity of pHLIPs for the phospholipid bilayer at low pH is 30–50 times higher than that at high pH. In addition, the kinetic process of insertion of pHLIPs into the cell membrane is quite rapid, and the movement from the formation of the interface helix to the transmembrane can be completed in seconds to minutes [86].

By utilizing this mechanism, Yu et al. developed pHLIP-coated hollow gold nanospheres (HauNS) containing chlorin e6(Ce6) by electrostatic approach, achieving desirable pH-driven controlled therapy [80]. As an efficient smart responsive delivery system, the HauNS-pHLIP-Ce6 could not only amplify the accumulation and retention effects in the tumor, but also induce enhanced photothermal therapy (PTT)/photodynamic therapy (PDT) simultaneously by a single NIR light. The pH-activated nanospheres were believed to be a promising theranostic platform against tumors. Additionally, Zhang et al. constructed mesoporous organosilica nanoparticles (MONs) modified with pHLIP (Figure 2C) [81], and these pHLIP-modified MONs loaded with DOX could achieve higher cellular uptake by MDA-MB-231 and MCF-7 cells and exert excellent cytotoxic effects against cancer cells in the low pH circumstances of the TME. The results indicated that the pHLIP-modified MONs could be employed as desirable nanocarriers for enhancing chemotherapy.

### 3.2. GSH-Responsive Nanocarriers

**Figure 3:**
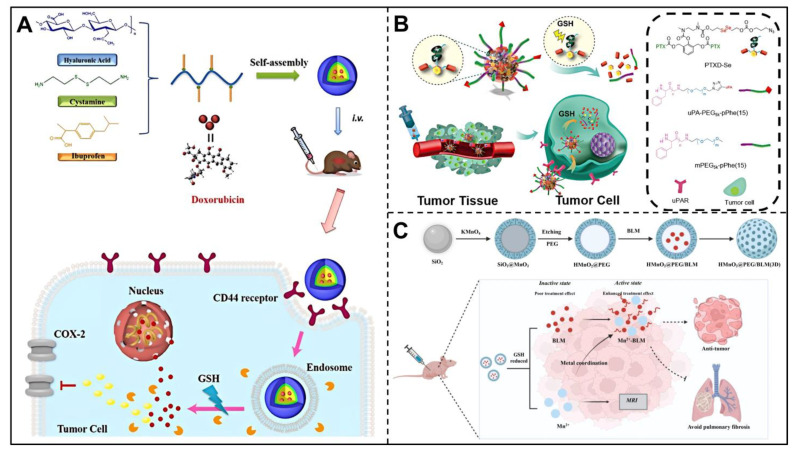
Design and responsive mechanism of GSH-triggered srNPs. (**A**) Preparation and in vivo release mechanism of HA-ss-BF. Reproduced with permission [88]. Copyright 2020, Elsevier. (**B**) Schematic showing composition and GSH-triggered drug release of uPA-PTXD NPs. Reproduced with permission [91]. Copyright 2018 Ivyspring International Publisher. (**C**) Diagram illustrating formulation and application of HMnO@PEG/BLM. Reproduced with permission [96]. Copyright 2022, American Chemical Society.

Overexpressed GSH in the TME can also serve as a trigger switch for tumor responsive therapy [87]. Disulfide bonds (-SS-), diselenide bonds (-SeSe-) and manganese dioxide (MnO_2_) can break or disintegrate when incubated with GSH, so they could be used to design intelligent nanoplatforms to achieve tumor-specific drug release. Disulfide bonds have been widely used as a breakable bonding bond in nanocarriers to make nano preparations GSH responsive. For example, Chai et al. designed the GSH-responsive micelles, hyaluronic acid-ibuprofen (HA-ss-BF), prepared by conjugating ibuprofen (BF) to hyaluronic acid (HA) with a disulfide bond and self-assembling into micelles [88]. HA-ss-BF micelles could be employed as stimuli-responsive carriers for delivering DOX. Specifically, BF is used as an anticancer agent inhibiting the overexpressed cyclooxygenase-2 (COX-2) in cancer cells. Furthermore, HA-ss-BF micelles loaded with DOX could target cancer cells by recognition of CD44 receptors. Thus, HA-ss-BF micelles achieved GSH-responsive disassembly, targeted and on-demand release of drugs, as well as improved cellular uptake and excellent biodistribution (Figure 3A). Liu et al. developed GSH-responsive and DNA-based branched nanoplatforms for codelivery of gene editing components sgRNA/Cas9 targeting DNA and gene silencing component antisense targeting mRNA [89]. To be specific, a 3′ terminal extended single guide RNA (sgRNA) was prepared, which is capable of complementary base pairing with complementary nucleic acid (antisense), and it was utilized to achieve coassembly of the sgRNA/Cas9/Antisense complex (RCA@NP) with antisense modified by two disulfide linkages as linker. The disulfide bonds enable RCA@NP to release antisense and sgRNA/Cas9 stepwise with the trigger of GSH and ribonuclease H enzymes (an intracellular RNA nuclease that can digest the RNA of RNA−DNA hybrids) in the intracellular environment, thus realizing synergistic antitumor efficacy. In addition, Ma et al. prepared ATRA-SS-HA nanomicelles by connecting hydrophobic all-trans retinoic acid (ATRA) and hydrophilic HA with disulfide bonds [90], which can self-assemble with curcumin (Cur) into Cur@ATRA-SS-HA. Such Cur-loaded nanomicelles can achieve GSH-triggered drug release due to the cleavage of disulfide bonds of nanomicelles in the TME, thus enhancing target esophageal cancer therapy.

Diselenide bonds also attract increasing attention as a GSH-responsive trigger. He et al. constructed diselenide-based GSH-responsive nanoparticles for triple-negative breast cancer-targeting (TNBC-targeting) treatment (Figure 3B) [91]. First, the paclitaxel (PTX) dimer prodrug PTXD-Se was synthesized with a diselenide bond serving as linkage, and it was subsequently encapsulated into an amphiphilic copolymer. As a ligand of urokinase-type plasminogen activator receptor (uPAR) that expresses highly in TNBC ligand, uPA peptide was modified on the PTXD-Se NPs surface to obtain the uPA-PTXD NPs for further TNBC-targeting treatment. Since the diselenide bonds could be responsively cleaved by high GSH concentration and the uPA could bind with uPAR, the uPA-PTXD NPs showed GSH-controlled drug release and targeted tumor accumulation, exhibited significant anti-tumor efficacy and reduced systemic toxicity. Although both disulfide and diselenide bonds are highly sensitive to GSH, there is still debate as to which molecule is more sensitive. He et al. reported that the GSH-responsiveness of diselenide bonds is more sensitive than that of disulfide bonds [92], while Zhang et al. [93,94] believed that disulfide bonds possessed stronger reduction sensitivity than disulfide bonds. In any case, these srNPs triggered by GSH show enhanced antitumor activity and reduced toxicity, which have broad development prospects.

Besides disulfide bonds and diselenide bonds, MnO_2_ is also could be utilized to design promising GSH-responsive nanocarriers. Mesoporous silica nanoparticles coated with MnO_2_ achieved GSH-responsive release as the MnO_2_ shell could be triggered to dissociate by abundant GSH in the TME, thus to deplete GSH and produce Fenton-like Mn^2+^ for cancer imaging and self-reinforced chemodynamic therapy (CDT) [95]. Zhang et al. constructed HMnO_2_@PEG/BLM nanoparticles, the polyethylene glycol (PEG)-modified hollow mesoporous MnO_2_ nanoparticles loading bleomycin (BLM) that needs to be activated by metal ions to exert cytotoxicity [96]. When the nanoparticles reached the TME, they were degraded by the excessive GSH and produced Mn^2+^, releasing BLM simultaneously, thereby forming Mn^2+^-BLM in situ and activating the therapeutic activity of BLM. In addition, Mn^2+^ could be utilized for in vivo magnetic resonance imaging (MRI). The nanoparticles effectively enhanced the antitumor therapeutic effects and avoided the adverse effects with GSH-responsive release and activation in situ of BLM (Figure 3C).

### 3.3. Hypoxia-Responsive Nanocarriers

**Figure 4:**
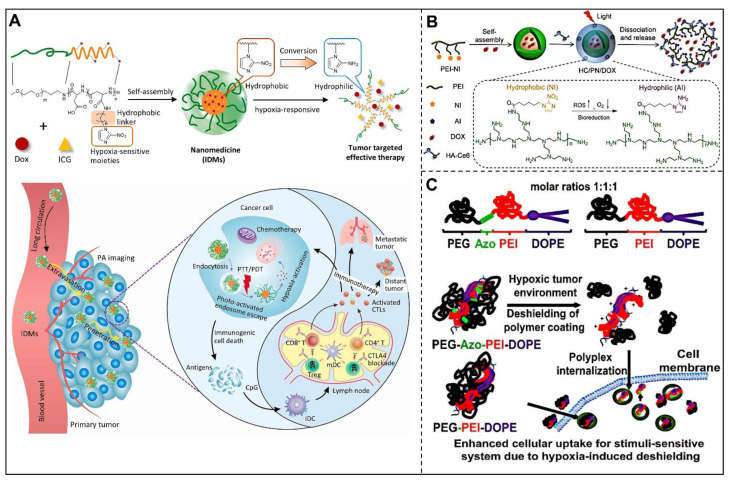
Design and responsive mechanism of hypoxia-triggered srNPs. (**A**) Preparation and in vivo selectively hypoxia-activated mechanism of IDMs nanomedicines for eradicating advanced breast cancer. Reproduced with permission [99]. Copyright 2021, Elsevier. (**B**) Chemical structure and hypoxia-responsive mechanism of NI-based nanoparticles. Reproduced with permission [98]. Copyright 2018, Royal Society of Chemistry. (**C**) Schematic illustration of synthesized polymers and their mechanism of hypoxia-responsive disintegration in the TME. Reproduced with permission [100]. Copyright 2014, Wiley-VCH GmbH.

Since hypoxia plays a key role in tumor angiogenesis, invasion, metastasis and immunosuppression [97], there has been increasing interest in developing nanocarriers for targeting the hypoxic TME recently. He et al. synthesized hypoxia-sensitive polyethylenimine-nitroimidazole (PEI-NI) micelles by self-assembly for codelivery of DOX and hyaluronic acid-Ce6 (HC) [98]. Under the hypoxic TME, the hydrophobic 2-nitroimidazole (NI) in micelles could be reduced to hydrophilic 2-aminoimidazole (AI) by a series of bio-reducing agents, enabling the micelles to release drugs by degradation to exert synergistic chemotherapy and PDT effects against cancer cells (Figure 4B). In addition, for accurate diagnosis and targeted treatment against tumors, Liu et al. constructed novel hypoxia-activatable polymeric micelles PEG-b-P(Asp-g-NIDH) consisting of 6-(2-nitroimidazole)hexylamine (NIDH) moieties grafted to PEG-b-poly (aspartic acid) (PEG-b-PAsp) for codelivery of ICG and DOX through self-assembly [99]. Owing to the presence of hypoxia-sensitive NIDH, PEG-b-P(Asp-g-NIDH) could achieve controlled drug release as well as synergistic chemotherapy and PTT/ PDT effects, facilitating precision of photoacoustic (PA) imaging and eradication of malignancy (Figure 4A).

Another common hypoxia-sensitive structure is azobenzene (AZO). Perche et al. developed nanocarriers for siRNA delivery by introducing AZO structures between PEG and polyethyleneimine (PEI) (Figure 4C) [100]. After reaching the oxygen-deficient TME, the AZO bond broke and led to the disappearance of the shielding effect of the PEG-coating and the release of the encapsulated siRNA. Yang et al. synthesized a human serum albumin (HSA)-based hypoxia-responsive nanoparticle HCHOA by crosslinking AZO with oxaliplatin prodrug-conjugated HSA(HO) and Ce6-conjugated HSA(HC) [101]. The AZO group could be cleaved under hypoxic conditions, causing the rapid hypoxia-induced degradation of HCHOA in the TME. The hypoxia-triggered disassembly mode of HCHOA ensures its enhanced intratumoral penetration and PDT performance. Kulkarni et al. constructed the novel vesicle by self-assembly of di-block copolymer polylactic acid-AZO-polyethylene glycol for loading anticancer drugs gemcitabine (GEM) and erlotinib (ERL) [102]. The results indicated that drug-loaded vesicles could release encapsulated drugs in a hypoxic environment and significantly inhibit the proliferation of pancreatic cancer cells. To increase the penetration of drugs, nucleic acids, or probes into the core of tumors, Xie et al. designed pH-sensitive and size-shrinkable nanocarrier PAMAM-AZO-PEG (PAP) [103], which can encapsulate DOX in the core and absorb HIF-1α siRNA (si-HIF) on the surface with electrostatic bonding. In order to monitor the anti-cancer effect of DOX, the ROS probe also was combined with PAP+DOX. In the hypoxic TME, the PEG detached from positively charged PAMAM owing to the hypoxia-induced cleavage of AZO, enabling PAMAM to escape from endosomes through the proton pump effect, thus releasing loaded DOX and si-HIF. The results of the assay demonstrated that PAP+si-HIF+DOX can promote DOX penetration and silence HIF-1α expression and detect the elevated ROS level induced by DOX.

### 3.4. Enzyme-Responsive Nanocarriers

According to the literature, there were several enzymes overexpressed in tumor cells that could be utilized as endogenous stimuli for cancer imaging and treatment [104,105]. The advantage of enzymes as a reaction trigger are their high specificity for substrates, arousing increasing interest in developing enzyme-induced nanocarriers. The main endogenous enzymes studied include cathepsin, matrix metalloproteinase, phospholipase, glycosidase and so on (Table 2).

#### 3.4.1. Cathepsin B

Cathepsin B (Cat-B) belongs to the family of lysosomal cysteine proteases, which are closely related to the development of cancer, and it is a typical stimulator, as its concentration in a variety of tumors is 3 to 9 times higher than that in normal tissues. Therefore, Cat-B-triggered srNPs have become new strategies for tumor treatment. Based on this, several Cat-B-cleavable peptides were designed, such as Glycine–Phenylalanine–Leucine–Glycine (GFLG) peptides [106,107] and Phenylalanine–Arginine–Arginine–Glycine (FRRG) peptides [108,109].

**Figure 5:**
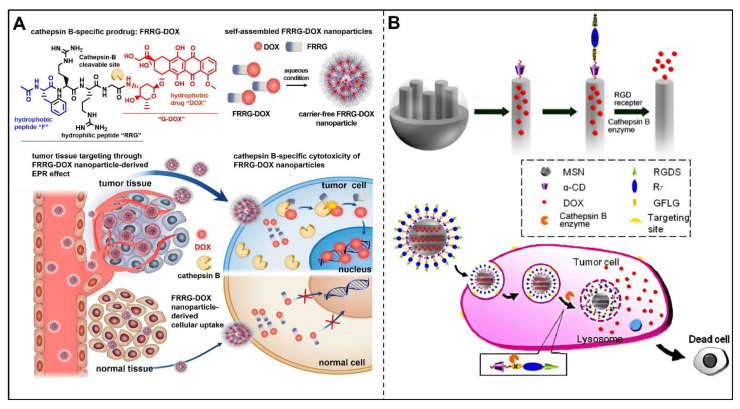
Design and responsive mechanism of Cat-B-triggered srNPs. (**A**) Schematic representation of construct of FRRG-DOX nanoparticles and its tumor-targeting cytotoxicity due to the Cat-B-specific enzyme. Reproduced with permission [108]. Copyright 2019, Elsevier. (**B**) Cat-B-induced procedure and targeting intracellular application of the MSNs. Reproduced with permission [106]. Copyright 2015, American Chemical Society.

For instance, Cheng et al. created enzyme-responsive MSNs with a rotaxane structure serving as gatekeeper on the orifices (Figure 5B) [106]. The MSNs subsequently were modified by a multifunctional peptide containing Cat-B-responsive GFLG, exhibiting a high encapsulation rate for DOX. Thus, the DOX-loaded MSNs are capable of rapidly releasing the drug when the GFLG peptide was cleaved by excessive cathepsin B in the TME, resulting in superior antitumor activity. Song et al. developed Cat-B-degradable peptide nanoparticles (Arg–His–(Gly–Phe–Lue–Gly)_3_ (RH–(GFLG)_3_) for delivery of DOX [110]. Compared to the control group, the DOX-loaded RH–(GFLG)_3_ exhibited enhanced stability, cell penetration and anti-cancer effects.

In addition, based on the Cat-B-cleavable peptide FRRG, Shim et al. designed a facile method for preparing the Cat-B-sensitive prodrug FRRG-DOX, which could release drugs with Cat-B-specific cleavage of prodrugs at the tumor site (Figure 5A) [108]. The results of human xenograft tumor models indicated that FRRG-DOX could improve targeting efficiency and antitumor efficacy against Cat-B-overexpressed cancer cells, and it did not cause severe toxicity in normal tissues owing to the low expression of Cat-B. Similarly, Cho et al. developed FRRG-monomethyl auristatin E (FRRG-MMAE) nanoparticles through self-aggregation [109], increasing the MMAE accumulation in tumors and enhancing the safety of therapy.

#### 3.4.2. Matrix Metalloproteinases

In recent years, MMPs have become a hot research target in cancer therapy. MMPs are proteolytic enzymes whose basic role is to promote protein degradation and participate in regulating a variety of cell behaviors related to cancer biology. They belong to the zinc and calcium-dependent endopeptidases family and are essential for tissue remodeling [111,112]. Under normal physiological conditions, the activity of MMPs is inhibited; However, in the TME, the abnormally high expression of some MMPs, such as MMP2, MMP7, MMP9 and so on, promotes the occurrence and metastasis of tumors [113]. Therefore, MMPs are widely used to achieve enzyme-triggered site-specific drug release. MMPs-responsive drug delivery can be achieved through constructing micelles, liposomes, dendrimers, nanogels and inorganic nanoparticles with MMPs-triggered structures [114]. Zhu et al. linked PTX with PEG_2000_ through an MMP2-responsive peptide, constructing a novel targeted nanomicelle [115]. Specifically, PTX was loaded into the hydrophobic core of the micelles and was covered with hydrophilic PEG. After intravenous administration, nanomicelles could accumulate at the tumor through the retention effect (EPR effect) and enhance permeability of solid tumors, and then they could disintegrate to release PTX under the action of MMP2 in TME. Additionally, Zhou et al. designed a strategy to integrate the OXA-prodrug hexadecyl-oxaliplatin diethylene amine with 2,3-dimethylmaleic anhydride (HOAD) and PEGylated photosensitizer in MMP-2-activatable prodrug vesicles (MPV) into a nanoplatform (MPV-HOAD) [116]. This nanoplatform remained stable in the blood circulation, while after reaching tumors, the PEG corona was removed by MMP-2 and its surface charge was transformed from negative to positive under the acidic TME, subsequently improving penetration and accumulation of drugs in tumors, thus achieving an excellent antitumor effect.

Kalafatovic et al. developed an MMP-9-sensitive amphiphilic peptide that can form micelles through self-assembly for loading DOX [117]. Notably, in the TME, the cleavage of the MMP-9-triggered linkage in peptides could exert micelle reconfiguring to fibrillar nanostructures due to catalyzed hydrolysis of MMP-9, thus releasing DOX slowly and continuously. In addition, Liu et al. constructed an MMP-13-responsive MSNs-PLGLAR-BSA-LA@DOX [118]. Specifically, PLGLAR, the MMP-13 substrate polypeptide sequence, was used as a responsive linkage, bovine serum albumin (BSA) was used as end-capping to seal the MSNs and lactobionic acid (LA) was used as a targeting ligand. The results demonstrated that DOX in functionalized MSNs could be effectively released under the trigger of MMP-13 in the TME, thus enabling the nano-drug treatment group to exhibit stronger efficacy and lower toxicity compared with the free DOX group.

#### 3.4.3. Phospholipase

Phospholipases can hydrolyze phospholipids into fatty acids and other lipophilic substances. In addition, as part of the host defense mechanism, phospholipase is overexpressed in the surrounding invasive region of some tumors [119], which provides a specific stimulus for responsive drug release. At present, the research on the phospholipase A2 (PLA2) family is most extensive, including intracellular PLA2 and secretory phospholipase A2 (sPLA2).

**Figure 6:**
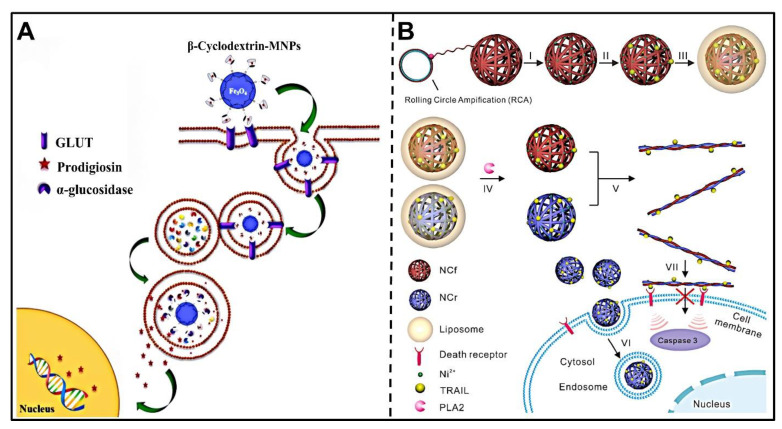
Design and responsive mechanism of enzyme-triggered srNPs. (**A**) Illustration of recognition, transport and α-glycosidase-triggered release of prodigiosin-loaded β-cyclodextrin-MNPs at the tumor site. Reproduced with permission [128]. Copyright 2017, Elsevier. (**B**) Illustration of synthesis and sPLA2-triggered activation mechanism of TRAIL-loaded DNA NCs covered with liposome. Reproduced with permission [120]. Copyright 2016, Elsevier.

Sun et al. designed sPLA2-degradable nanoparticles consisting of a liposome as the shell and two complementary cytokine-loaded DNA nanoclews (NCs) as cores [120]. Specifically, DNA NC cores were loaded with the tumor necrosis factor-related apoptosis-inducing ligand (TRAIL), a model cytokine, through Ni^2+^-polyhistidine affinity between Ni^2+^-modified DNA NCs and TRAIL, and the sPLA2-triggered liposome shell could protect the TRAIL-loaded DNA NC cores from degradation in the physiological environment and could be degraded by overexpressed sPLA2 in the TME. Thus, TRAIL-loaded DNA NCs could transform into nanofibers extracellularly and deliver TRAIL to death receptors on the plasma membrane of cancer cells, thereby activating the apoptotic signal (Figure 6B). The Andresen group developed an enzymatically-triggered prodrug liposome for delivering antitumor ether lipids (AELs) to tumor sites, and the liposomes also could be utilized to encapsulate various chemotherapeutics [121]. To be specific, sPLA2-sensitive masked AELs (proAELs) were synthesized, which could form liposomal membranes spontaneously in an aqueous solution. In the TME, owing to overexpressed sPLA2, the hydrolysis of ester bonds in proAELs led to the disintegration of liposomes, releasing activated cytotoxic AELs and free fatty acids/encapsulated drugs. In another study, Lee et al. designed a strategy for the synthesis of sPLA2-sensitive phosphate micelles loaded with up-conversion nanoparticles (UCNP) [122], which could achieve targeted delivery of UCNPs to prostate cancer cells for accurate bio-imaging. The biocompatible UCNP-loaded micelles exhibited precise drug release and reduced adverse effects. Ghavami et al. constructed sPLA2-responsive DSPC/DSPG/DSPE-PEG_2000_ liposomes for targeted delivery of peptide nucleic acid (PNA), an antisense agent [123], to the tumor. Specifically, antisense octaarginine (a cell-penetrating peptide)-conjugated PNA (octaarginine-PNA) was prepared and encapsulated into the liposome. The PNA-loaded liposomes showed efficient sPLA2-induced release and excellent antisense effects against HeLa cells.

#### 3.4.4. Glycosidases

Glycosidases participate in the occurrence of N-linked glycosylation in the Golgi apparatus and endoplasmic reticulum [124], and they also can hydrolyze carbohydrates in the lysosomes [125]. Thus, glycosidases could be utilized to design glycosidase-triggered nanocarriers for delivering drugs to the target tissues with high concentrations of glycosidases. In recent years, researchers have developed various glycosylated nanoparticles to selectively release drugs in the TME where cancer cells overexpress glycosidases. Bernardos et al. developed silica mesoporous supports (SMPS) modified with lactose or starch derivatives on the surface of nanoparticles to achieve enzyme-induced drug release [126]. The fluorescent dyes were effectively blocked in the pores of SMPS by the grafted saccharide molecules. With the triggering of β-D-galactosidase, the coated saccharides of MSNs were hydrolyzed and the entrapped dye was released. For efficient drug delivery, Clarhaut et al. synthesized a β-galactosidase-sensitive folate-DOX conjugate (FDC) consisting of a folate ligand, DOX and a galactoside trigger, which can selectively recognize folate receptor-positive acute myeloid leukemia (AML) cells and release the DOX due to the carbohydrate unit of FDC being degraded by the catalysis of intracellular β-galactosidase [127]. Rastegari et al. constructed Fe_3_O_4_ magnetic nanoparticles (β-CD-MNPs) coated with β-cyclodextrin (β-CD) that were employed as enzyme-sensitive carriers for delivering prodigiosin (PG) to cancer cells (Figure 6A) [128]. The PG-loaded β-CD-MNPs were responsive to galactosidase, releasing drugs due to α-glucosidase degradation, thereby realizing targeted drug release and improved drug retention to MCF-7/GFP and HepG2 cells.

**Table 2:** Enzyme-responsive srNPs in cancer therapy.

Stimulus	Responsive Moiety	Nanoplatform	Cargos	Application	Tumor Model	Refs.
Cathepsin B	GFLG	DOX@MSN-GFLGR7RGDS/α-CD	DOX	Tumor targeted chemotherapy	HeLa	[106]
		RH-(GFLG)_3_	DOX	Tumor targeted chemotherapy	HeLa	[110]
	FRRG	FRRG-DOX	DOX	Tumor targeted chemotherapy	HT-29	[108]
		FRRG-MMAE	MMAE	Tumor targeted chemotherapy	4T1	[109]
MMP-2	GPLGIAGQ	PEG_2000_-peptide-PTX	PTX	MMP-2-sensitive drug release	A549	[115]
	GPLGLAG	MPV-HOAD	OXA pheophorbide a	MMP-2-sensitive PDT and cancer immunotherapy	CT26	[116]
MMP-9	GFFLG PhAc-FFAG	MMP-9 responsive peptides in conjunction with DOX	DOX	MMP-9-triggered drug release and chemotherapy	MDA-MB-231-luc-D3H2LN	[117]
MMP-13	PLGLAR	MSNs-PLGLAR-BSA-LA@DOX	DOX	MMP-13-triggered drug release and chemotherapy	HepG2	[118]
sPLA2	1-palmitoyl-2-oleoyl-sn-glycero-3-phosphocholine	liposome shells and TRAIL-loaded DNA NCs cores	TRAIL	Targeted Delivery of Cytokine	COLO 205 cells	[120]
	ester bonds in proAEL	proAEL	AEL	Tumor specific drug release for cancer therapy	KATO III	[121]
	phosphate	UCNP-loaded phosphate micelles	UCNP	Bioimaging of prostate cancer cells	22Rv1	[122]
	DSPC/DSPG/DSPE	DSPC/DSPG/DSPE liposomes	PNA	tumor targeted drug release for cancer therapy	Hela	[123]
Galactosidase	Saccharides	SMPS modified with lactose or starch derivatives	[Ru(bipy)_3_]^2+^ dye	Glycosidase-responsive intracellular controlled release of drug	HeLa	[126]
Galactosidase	Carbohydrate unit	folate-DOX conjugate	DOX	Glycosidase-responsive chemotherapy	KG-1 and HL-60	[127]
α-glucosidase	β-CD	β-CD-MNPs	prodigiosin	Anticancer drug delivery	MCF-7/GFPHepG2	[128]

### 3.5. ROS-Responsive Nanocarriers

**Figure 7:**
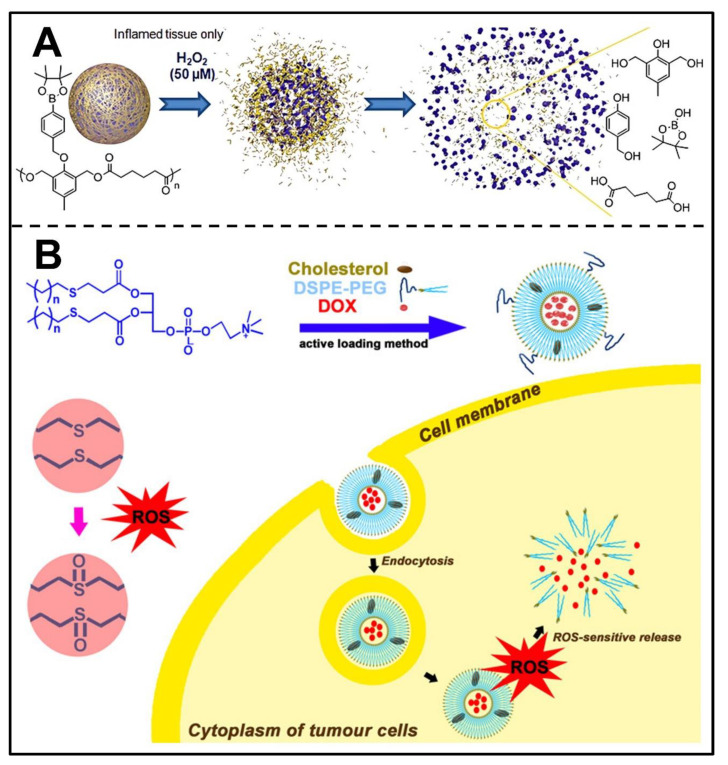
Design and responsive mechanism of ROS-triggered srNPs. (**A**) Composite and H_2_O_2_-degradable mechanism of polymeric nanoparticles. Reproduced with permission [133]. Copyright 2012, American Chemical Society. (**B**) Formulation and ROS-induced DOX release of S-LPs. Reproduced with permission [136]. Copyright 2019, American Chemical Society.

The higher level of ROS in cancer cells could also be used for the design and development of srNPs to enhance drug release at specific sites. In the design of ROS-responsive NDDS, the most commonly used groups are boric acid esters [129], thioketals [130], thioethers [131,132] and so on. A boric acid ester bond has been proven to be able to undergo ROS-induced degradation and its application in different fields has increased recently. For example, Lux et al. developed a novel ROS-responsive polyester containing a boronic ester that could degrade and release cargos through H_2_O_2_-triggered cleavage of the boronic ester (Figure 7A) [133]. Sun et al. designed a self-amplified ROS-induced D-α-tocopherol PEG_1000_ succinate-tamoxifen (TPGS-TAM) with an aryl boronic ester linker [134]. After internalization by cancer cells, such a conjugate can disintegrate to release TAM and α-tocopherol succinate (α-TOS) that can subsequently increase ROS and further accelerate the release of TAM, thus achieving a robust anti-cancer effect.

Besides the boronic ester structure, thioketal was also used to design ROS-sensitive nanocarriers. Wang et al. constructed thioketal-core ROS-sensitive PAMAM dendrimers for loading siRNA(siRNA/ROS-PAMAM) [135], which can improve the release of siRNA in the TME. With thioketal employed as linkages, siRNA/ROS-PAMAM is cleavable in ROS-abundant conditions, reducing cytotoxicity for normal tissues. The experimental results demonstrated that siRNA/ROS-PAMAM shows high gene transfection efficiency for A549 cells.

Thioethers have also been utilized to design ROS-triggered srNPs. Du et al. synthesized novel thioether phosphatidylcholines (S-PCs) and S-PC-based liposomes (S-LPs) loaded with DOX for ROS-induced release of drugs (Figure 7B) [136]. The results of in vitro and in vivo assays proved that S-LPs exhibited efficient ROS-responsive targeted drug release due to thioether oxidation, thereby enhancing the potency of antitumor drugs.

## 4. Conclusions and Perspectives

Tumor growth shows cellular and molecular heterogeneity [137], and it has been reported that a small quantity of progenitor cells and stem cells embedded in the perivascular region might be related to the growth and recurrence of tumors [138]. At the cellular level, malignant tumors are characterized by a complex mixture of benign cells, malignant cells, fibroblasts, stromal cells, vascular cells and infiltrating inflammatory cells. At the molecular level, the phenotype and gene expression profile of cancer cells are distinctly different from those of normal cells. This review comprehensively summarizes a series of intelligent nanoplatforms responsive to specific stimuli in the TME, including low pH, high GSH concentration, hypoxia, overexpressed enzymes and excessive ROS. Notably, the srNPs’ target tumor tissues and can be triggered by endogenous stimulation of the TME, thus showing enhanced anti-tumor efficacy and reduced toxicity. Additionally, the srNPs play a crucial role in drug controlled-release against cancer cells as the active participant instead of the passive carrier, showing great development potential in improving disease treatment methods.

Compared with conventional drug delivery systems, TME-responsive nanoformulations control drug uptake and release in cancer cells and the TME through local stimulation, exerting excellent antitumor therapeutic efficacy. However, there are still some key problems to be solved before the srNPs are applied in clinical practice. Herein, we highlight some obstacles that must be eliminated as soon as possible and provide some suggestions for future applications.

Firstly, conditions of endogenous stimuli in some studies are imprecise, for example, the pH value used in some studies is lower than the actual pH of the tumor, and the concentration of the reducing agent used in vitro is higher than the actual concentration in vivo. Therefore, a better understanding of the differences between the normal physiological environment and the TME is essential to further develop stimuli-responsive nanocarriers. In addition, variability among patients and differences in the TME among diverse types of tumors hindered clinical translation of srNPs. To remove the obstacles caused by tumor heterogeneity, more triggers that are overexpressed in TME should be found and studied, such as vascular endothelial growth factor (VEGF) [139], and multi-stimuli-responsive nanocarriers with tunable drug delivery should be developed to adapt to different tumors and different patients. In addition, exogenous stimuli [140], such as heat, light and ultrasound, also play an important role in targeted anti-tumor therapy, so research on these stimuli should also be promoted and explored in the future.

Secondly, numerous studies on srNPs in subcutaneous tumor-bearing mice models have been reported, but their therapeutic effect can rarely predict their safety and effectiveness in clinical trials [141]. Thus, more preclinical animal models that could highly simulate human TME need to be established, such as patient-derived tumor models [142], tumor metastasis models, in situ tumor models and tumor drug resistance models to further study the possible clinical applications of srNPs.

Thirdly, the potential systemic toxicity of srNPs is a severe challenge for their use in long-term treatments due to some non-biodegradable nanomaterials and relatively low accumulation efficiency at the target site. To overcome this obstacle, some strategies could be utilized to verify the biosafety of srNPs in the short term and in the long term. A promising approach is to develop metabolizable or biodegradable nanomaterials for the design of functional srNPs based on artificial intelligence and machine learning to avoid systemic toxicity [143]. In addition, a series of studies need to be comprehensively performed, such as the distribution of drugs intratumourally, scientific analysis of pharmacokinetics and pharmacodynamics, analysis of blood biochemistry and hematology, long-term toxicological evaluation and so forth [144].

Lastly, the sophisticated approaches and complex components for preparing srNPs severely hampered their clinical application due to the high cost, batch-to-batch variations and relatively poor stability of srNPs. The simple and feasible fabrication methods for srNPs should be innovated to ensure controllable cost, batch-to-batch reproducibility and maintained stability, thus facilitating the standardized large-scale production and quality control of srNPs for clinical translation.

To summarize, srNPs-responsive nanocarriers represent a promising future for the treatment of cancer. We firmly believe that in the near future, with the breakthrough of relevant research, multifunctional nano delivery systems for practical clinical applications will make great contributions to human health.

## Data Availability

Not applicable.

## References

[1] Dai Y., Xu C., Sun X., Chen X. (2017). Nanoparticle design strategies for enhanced anticancer therapy by exploiting the tumour microenvironment. Chem. Soc. Rev..

[2] Sung H., Ferlay J., Siegel R.L., Laversanne M., Soerjomataram I., Jemal A., Bray F. (2021). Global cancer statistics 2020: GLOBOCAN estimates of incidence and mortality worldwide for 36 cancers in 185 countries. CA A Cancer J. Clin..

[3] Qin S.-Y., Cheng Y.-J., Lei Q., Zhang A.-Q., Zhang X.-Z. (2018). Combinational strategy for high-performance cancer chemotherapy. Biomaterials.

[4] Balkwill F.R., Capasso M., Hagemann T. (2012). The tumor microenvironment at a glance. J. Cell Sci..

[5] Yang S., Gao H. (2017). Nanoparticles for modulating tumor microenvironment to improve drug delivery and tumor therapy. Pharmacol. Res..

[6] Gong F., Yang N., Wang X., Zhao Q., Chen Q., Liu Z., Cheng L. (2020). Tumor microenvironment-responsive intelligent nanoplatforms for cancer theranostics. Nano Today.

[7] Helmlinger G., Sckell A., Dellian M., Forbes N.S., Jain R.K. (2002). Acid Production in Glycolysis-impaired Tumors Provides New Insights into Tumor Metabolism. Clin. Cancer Res..

[8] De Milito A., Iessi E., Logozzi M., Lozupone F., Spada M., Marino M.L., Federici C., Perdicchio M., Matarrese P., Lugini L. (2007). Proton Pump Inhibitors Induce Apoptosis of Human B-Cell Tumors through a Caspase-Independent Mechanism Involving Reactive Oxygen Species. Cancer Res..

[9] Vander Heiden M.G., Cantley L.C., Thompson C.B. (2009). Understanding the Warburg effect: The metabolic requirements of cell proliferation. Science.

[10] Gatenby R.A., Gillies R.J., Gatenby R.A., Gillies R.J. (2004). Why do cancers have high aerobic glycolysis?. Nat. Rev. Cancer.

[11] Sabharwal S.S., Schumacker P.T. (2014). Mitochondrial ROS in cancer: Initiators, amplifiers or an Achilles’ heel?. Nat. Rev. Cancer.

[12] Wu W., Luo L., Wang Y., Wu Q., Dai H.-B., Li J.-S., Durkan C., Wang N., Wang G.-X. (2018). Endogenous pH-responsive nanoparticles with programmable size changes for targeted tumor therapy and imaging applications. Theranostics.

[13] Wojtkowiak J.W., Verduzco D., Schramm K.J., Gillies R.J. (2011). Drug Resistance and Cellular Adaptation to Tumor Acidic pH Microenvironment. Mol. Pharm..

[14] Cheng R., Feng F., Meng F., Deng C., Feijen J., Zhong Z. (2011). Glutathione-responsive nano-vehicles as a promising platform for targeted intracellular drug and gene delivery. J. Control. Release.

[15] Fernandes P.A., Ramos M.J. (2004). Theoretical Insights into the Mechanism for Thiol/Disulfide Exchange. Chem.-A Eur. J..

[16] Estrela J.M., Ortega A., Obrador E. (2006). Glutathione in cancer biology and therapy. Crit. Rev. Clin. Lab..

[17] Thambi T., Deepagan V.G., Yoon H.Y., Han H.S., Kim S.H., Son S., Jo D.G., Ahn C.H., Suh Y.D., Kim K. (2014). Hypoxia-responsive polymeric nanoparticles for tumor-targeted drug delivery. Biomaterials.

[18] Lu Y., Aimetti A.A., Langer R., Gu Z. (2016). Bioresponsive materials. Nat. Rev. Mater..

[19] Tao J., Yang G., Zhou W., Qiu J., Chen G., Luo W., Zhao F., You L., Zheng L., Zhang T. (2021). Targeting hypoxic tumor microenvironment in pancreatic cancer. J. Hematol. Oncol..

[20] Cowman S.J., Koh M.Y. (2022). Revisiting the HIF switch in the tumor and its immune microenvironment. Trends Cancer.

[21] Kumari R., Sunil D., Ningthoujam R.S. (2020). Hypoxia-responsive nanoparticle based drug delivery systems in cancer therapy: An up-to-date review. J. Control. Release.

[22] Xu X.-X., Chen S.-Y., Yi N.-B., Li X., Chen S.-L., Lei Z., Cheng D.-B., Sun T. (2022). Research progress on tumor hypoxia-associative nanomedicine. J. Control. Release.

[23] Li Y., Gao J., Zhang C., Cao Z., Cheng D., Liu J., Shuai X. (2017). Stimuli-Responsive Polymeric Nanocarriers for Efficient Gene Delivery. Top. Curr. Chem..

[24] Qiu N., Gao J., Liu Q., Wang J., Shen Y. (2018). Enzyme-Responsive Charge-Reversal Polymer Mediated Effective Gene Therapy for Intraperitoneal Tumors. Biomacromolecules.

[25] Lee S.H., Gupta M.K., Bang J.B., Bae H., Sung H.J. (2013). Current progress in Reactive Oxygen Species (ROS)-Responsive materials for biomedical applications. Adv. Healthc. Mater..

[26] D’Autréaux B., Toledano M.B. (2007). ROS as signalling molecules: Mechanisms that generate specificity in ROS homeostasis. Nat. Rev. Mol. Cell Biol..

[27] Peng X., Gandhi V. (2012). ROS-activated anticancer prodrugs: A new strategy for tumor-specific damage. Ther. Deliv..

[28] Zhai S., Hu X., Hu Y., Wu B., Xing D. (2017). Visible light-induced crosslinking and physiological stabilization of diselenide-rich nanoparticles for redox-responsive drug release and combination chemotherapy. Biomaterials.

[29] Trachootham D., Alexandre J., Peng H., Trachootham D., Alexandre J., Huang P. (2009). Targeting cancer cells by ROS-mediated mechanisms: A radical therapeutic approach?. Nat. Rev. Drug Discov..

[30] Rao N.V., Ko H., Lee J., Park J.H. (2018). Recent Progress and Advances in Stimuli-Responsive Polymers for Cancer Therapy. Front. Bioeng. Biotechnol..

[31] Uthaman S., Huh K.M., Park I.-K. (2018). Tumor microenvironment-responsive nanoparticles for cancer theragnostic applications. Biomater. Res..

[32] Du J., Lane L.A., Nie S. (2015). Stimuli-responsive nanoparticles for targeting the tumor microenvironment. J. Control. Release.

[33] Zhou K., Wang Y., Huang X., Luby-Phelps K., Sumer B.D., Gao J. (2011). Tunable, Ultrasensitive pH-Responsive Nanoparticles Targeting Specific Endocytic Organelles in Living Cells. Angew. Chem..

[34] Du J.Z., Mao C.Q., Yuan Y.Y., Yang X.Z., Wang J. (2014). Tumor extracellular acidity-activated nanoparticles as drug delivery systems for enhanced cancer therapy. Biotechnol. Adv..

[35] Andreev O.A., Engelman D.M., Reshetnyak Y.K. (2014). Targeting diseased tissues by pHLIP insertion at low cell surface pH. Front. Physiol..

[36] Wong A.S.M., Mann S.K., Czuba E., Sahut A., Liu H., Suekama T.C., Bickerton T., Johnston A.P.R., Such G.K. (2015). Self-assembling dual component nanoparticles with endosomal escape capability. Soft Matter.

[37] Gunawan S.T., Liang K., Such G.K., Johnston A.P., Leung M.K., Cui J., Caruso F. (2014). Engineering enzyme-cleavable hybrid click capsules with a pH-sheddable coating for intracellular degradation. Small.

[38] Bilalis P., Tziveleka L.-A., Varlas S., Iatrou H. (2016). pH-Sensitive nanogates based on poly(L-histidine) for controlled drug release from mesoporous silica nanoparticles. Polym. Chem..

[39] Wu H., Zhu L., Torchilin V.P. (2013). pH-sensitive poly(histidine)-PEG/DSPE-PEG co-polymer micelles for cytosolic drug delivery. Biomaterials.

[40] Lee E.S., Oh K.T., Kim D., Youn Y.S., Bae Y.H. (2007). Tumor pH-responsive flower-like micelles of poly (L-lactic acid)-b-poly (ethylene glycol)-b-poly (L-histidine). J. Control. Release.

[41] Yin H., Lee E.S., Kim D., Lee K.H., Oh K.T., Bae Y.H. (2008). Physicochemical characteristics of pH-sensitive poly (L-histidine)-b-poly (ethylene glycol)/poly (L-lactide)-b-poly (ethylene glycol) mixed micelles. J. Control. Release.

[42] Lee E.S., Gao Z., Kim D., Park K., Kwon I.C., Bae Y.H. (2008). Super pH-sensitive multifunctional polymeric micelle for tumor pHe specific TAT exposure and multidrug resistance. J. Control. Release.

[43] Kim D., Lee E.S., Oh K.T., Gao Z.G., Bae Y.H. (2008). Doxorubicin-loaded polymeric micelle overcomes multidrug resistance of cancer by double-targeting folate receptor and early endosomal pH. Small.

[44] Lee E.S., Na K., Bae Y.H. (2005). Doxorubicin loaded pH-sensitive polymeric micelles for reversal of resistant MCF-7 tumor. J. Control. Release.

[45] Lee E.S., Na K., Bae Y.H. (2005). Super pH-sensitive multifunctional polymeric micelle. Nano Lett..

[46] Shi M., Zhao X., Zhang J., Pan S., Yang C., Wei Y., Hu H., Qiao M., Chen D., Zhao X. (2018). pH-responsive hybrid nanoparticle with enhanced dissociation characteristic for siRNA delivery. Int. J. Nanomed..

[47] Shi M., Zhang J., Huang Z., Chen Y., Pan S., Hu H., Qiao M., Chen D., Zhao X. (2020). Stimuli-responsive release and efficient siRNA delivery in non-small cell lung cancer by a poly (l-histidine)-based multifunctional nanoplatform. J. Mater. Chem. B.

[48] Liu M., Huang L., Zhang W., Wang X., Geng Y., Zhang Y., Wang L., Zhang W., Zhang Y.-J., Xiao S. (2022). A transistor-like pH-sensitive nanodetergent for selective cancer therapy. Nat. Nanotechnol..

[49] Yang X.-L., Wu W.-X., Li J., Hu Z.-E., Wang N., Yu X.-Q. (2020). A facile strategy to construct fluorescent pH-sensitive drug delivery vehicle. Polymer.

[50] Zhang S., Lv J., Gao P., Feng Q., Wang H., Cheng Y. (2021). A pH-responsive phase-transition polymer with high serum stability in cytosolic protein delivery. Nano Lett..

[51] Sethuraman V.A., Na K., Bae Y.H. (2006). pH-responsive sulfonamide/PEI system for tumor specific gene delivery: An in vitro study. Biomacromolecules.

[52] Kang H.C., Bae Y.H. (2007). pH-tunable endosomolytic oligomers for enhanced nucleic acid delivery. Adv. Funct. Mater..

[53] Zhang M., Liu J., Kuang Y., Li Q., Zheng D.-W., Song Q., Chen H., Chen X., Xu Y., Li C. (2017). Ingenious pH-sensitive dextran/mesoporous silica nanoparticles based drug delivery systems for controlled intracellular drug release. Int. J. Biol. Macromol..

[54] Huang L., Tao K., Liu J., Qi C., Xu L., Chang P., Gao J., Shuai X., Wang G., Wang Z. (2016). Design and fabrication of multifunctional sericin nanoparticles for tumor targeting and pH-responsive subcellular delivery of cancer chemotherapy drugs. ACS Appl. Mater. Interfaces.

[55] Thambi T., Deepagan V.G., Chang K.Y., Park J.H. (2011). Synthesis and physicochemical characterization of amphiphilic block copolymers bearing acid-sensitive orthoester linkage as the drug carrier. Polymer.

[56] Zha Q., Wang X., Cheng X., Fu S., Yang G., Yao W., Tang R. (2017). Acid–degradable carboxymethyl chitosan nanogels via an ortho ester linkage mediated improved penetration and growth inhibition of 3-D tumor spheroids in vitro. Mater. Sci. Eng. C.

[57] Belali S., Karimi A.R., Hadizadeh M. (2018). Cell-specific and pH-sensitive nanostructure hydrogel based on chitosan as a photosensitizer carrier for selective photodynamic therapy. Int. J. Biol. Macromol. Struct. Funct. Interact..

[58] Tao Y., Liu S., Zhang Y., Chi Z., Xu J. (2018). A pH-Responsive polymer based on dynamic imine bonds as a drug delivery material with pseudo target release behavior. Polym. Chem..

[59] Suarez S.L., Muñoz A., Mitchell A.C., Braden R.L., Luo C., Cochran J.R., Almutairi A., Christman K.L. (2016). Degradable acetalated dextran microparticles for tunable release of an engineered hepatocyte growth factor fragment. ACS Biomater. Sci. Eng..

[60] Shim M.S., Kim C.S., Ahn Y.-C., Chen Z., Kwon Y.J. (2010). Combined multimodal optical imaging and targeted gene silencing using stimuli-transforming nanotheragnostics. J. Am. Chem. Soc..

[61] Deirram N., Zhang C., Kermaniyan S.S., Johnston A.P., Such G.K. (2019). pH-responsive polymer nanoparticles for drug delivery. Macromol. Rapid Commun..

[62] Etrych T., Jelinkova M.A., Ríhová B., Ulbrich K. (2001). New HPMA copolymers containing doxorubicin bound via pH-sensitive linkage: Synthesis and preliminary in vitro and in vivo biological properties. J. Control. Release.

[63] Chytil P., Koziolová E., Etrych T., Ulbrich K. (2018). HPMA Copolymer–Drug Conjugates with Controlled Tumor-Specific Drug Release. Macromol. Biosci..

[64] Zhou Z., Li L., Yang Y., Xu X., Huang Y. (2014). Tumor targeting by pH-sensitive, biodegradable, cross-linked N-(2-hydroxypropyl) methacrylamide copolymer micelles. Biomaterials.

[65] Liao J., Zheng H., Fei Z., Lu B., Zheng H., Li D., Xiong X., Yi Y. (2018). Tumor-targeting and pH-responsive nanoparticles from hyaluronic acid for the enhanced delivery of doxorubicin. Int. J. Biol. Macromol..

[66] Huang X., Du F., Cheng J., Dong Y., Liang D., Ji S., Lin S.-S., Li Z. (2009). Acid-sensitive polymeric micelles based on thermoresponsive block copolymers with pendent cyclic orthoester groups. Macromolecules.

[67] Huang X., Du F., Ju R., Li Z. (2007). Novel acid-labile, Thermoresponsive poly (methacrylamide) s with pendent Ortho Ester moieties. Macromol. Rapid Commun..

[68] Xu Z., Lai J., Tang R., Ji W., Wang R., Wang J., Wang C. (2014). Synthesis and Characterization of Homopolymers Bearing Acid-Cleavable Cationic Side-Chains for pH-Modulated Release of DNA. Macromol. Biosci..

[69] Li Y., Song L., Lin J., Pan Z., Ma J., Zhang Y., Su G., Ye S., Luo F.H., Zhu X. (2017). Programmed Nanococktail Based on pH-Responsive Function Switch for Self-Synergistic Tumor-Targeting Therapy. Acs Appl. Mater. Interfaces.

[70] Feng X., Li D., Han J., Zhuang X., Ding J. (2017). Schiff base bond-linked polysaccharide–doxorubicin conjugate for upregulated cancer therapy. Mater. Sci. Eng. C.

[71] Liao S.-C., Ting C.-W., Chiang W.-H. (2020). Functionalized polymeric nanogels with pH-sensitive benzoic-imine cross-linkages designed as vehicles for indocyanine green delivery. J. Colloid Interface Sci..

[72] Gillies E.R., Goodwin A.P., Fréchet J.M.J. (2004). Acetals as pH-sensitive linkages for drug delivery. Bioconjugate Chem..

[73] Wagner J., Gößl D.E., Ustyanovska N., Xiong M., Hauser D., Zhuzhgova O., Hocevar S., Taskoparan B.L., Poller L., Datz S. (2021). Mesoporous silica nanoparticles as pH-responsive carrier for the immune-activating drug resiquimod enhance the local immune response in mice. ACS Nano.

[74] Bachelder E.M., Beaudette T.T., Broaders K.E., Dashe J., Fréchet J.M. (2008). Acetal-Derivatized Dextran: An Acid-Responsive Biodegradable Material for Therapeutic Applications. J. Am. Chem. Soc..

[75] Zhang Z., Chen X., Chen L., Yu S., Cao Y., He C., Chen X. (2013). Intracellular pH-sensitive PEG-block-acetalated-dextrans as efficient drug delivery platforms. ACS Appl. Mater. Interfaces.

[76] Cohen J.A., Beaudette T.T., Cohen J.L., Broaders K.E., Bachelder E.M., Frechet J.M.J. (2010). Acetal-Modified Dextran Microparticles with Controlled Degradation Kinetics and Surface Functionality for Gene Delivery in Phagocytic and Non-Phagocytic Cells. Adv. Mater..

[77] Bachelder E.M., Pino E.N., Ainslie K.M. (2017). Acetalated dextran: A tunable and acid-labile biopolymer with facile synthesis and a range of applications. Chem. Rev..

[78] Cui L., Cohen J.L., Chu C.K., Wich P.R., Kierstead P.H., FréChet J.M. (2012). Conjugation chemistry through acetals toward a dextran-based delivery system for controlled release of siRNA. J. Am. Chem. Soc..

[79] Ornelas-Megiatto C., Shah P.N., Wich P.R., Cohen J.L., Tagaev J.A., Smolen J.A., Wright B.D., Panzner M.J., Youngs W.J., Fréchet J.M.J. (2012). Aerosolized antimicrobial agents based on degradable dextran nanoparticles loaded with silver carbene complexes. Mol. Pharm..

[80] Yu M., Guo F., Wang J., Tan F., Li N. (2016). A pH-Driven and photoresponsive nanocarrier: Remotely-controlled by near-infrared light for stepwise antitumor treatment. Biomaterials.

[81] Zhang Y., Dang M., Tian Y., Zhu Y., Liu W., Tian W., Su Y., Ni Q., Xu C., Lu N. (2017). Tumor acidic microenvironment targeted drug delivery based on pHLIP-modified mesoporous organosilica nanoparticles. ACS Appl. Mater. Interfaces.

[82] Weerakkody D., Moshnikova A., Thakur M.S., Moshnikova V., Daniels J., Engelman D.M., Andreev O.A., Reshetnyak Y.K. (2013). Family of pH (low) insertion peptides for tumor targeting. Proc. Natl. Acad. Sci. USA.

[83] Wyatt L.C., Lewis J.S., Andreev O.A., Reshetnyak Y.K., Engelman D.M. (2017). Applications of pHLIP technology for cancer imaging and therapy. Trends Biotechnol..

[84] Reshetnyak Y.K., Andreev O.A., Lehnert U., Engelman D.M. (2006). Translocation of molecules into cells by pH-dependent insertion of a transmembrane helix. Proc. Natl. Acad. Sci. USA.

[85] Thévenin D., An M., Engelman D.M. (2009). pHLIP-Mediated Translocation of Membrane-Impermeable Molecules into Cells. Chem. Biol..

[86] Andreev O.A., Karabadzhak A.G., Weerakkody D., Andreev G.O., MEngelman D., Reshetnyak Y.K. (2010). pH (low) insertion peptide (pHLIP) inserts across a lipid bilayer as a helix and exits by a different path. Proc. Natl. Acad. Sci. USA.

[87] Zhao J., Yang Y., Han X., Liang C., Liu J., Song X., Ge Z., Liu Z. (2017). Redox-Sensitive Nanoscale Coordination Polymers for Drug Delivery and Cancer Theranostics. Acs Appl. Mater. Interfaces.

[88] Chai Z., Teng C., Yang L., Ren L., Yuan Z., Xu S., Cheng M., Wang Y., Yan Z., Qin C. (2020). Doxorubicin delivered by redox-responsive Hyaluronic Acid–Ibuprofen prodrug micelles for treatment of metastatic breast cancer. Carbohydr. Polym..

[89] Liu J., Wu T., Lu X., Wu X., Liu S., Zhao S., Xu X., Ding B. (2019). A self-assembled platform based on branched DNA for sgRNA/Cas9/antisense delivery. J. Am. Chem. Soc..

[90] Ma Z., Gao X., Raza F., Zafar H., Huang G., Yang Y., Shi F., Wang D., He X. (2022). Design of GSH-Responsive Curcumin Nanomicelles for Oesophageal Cancer Therapy. Pharmaceutics.

[91] He X., Zhang J., Li C., Zhang Y., Lu Y., Zhang Y., Liu L., Ruan C., Chen Q., Chen X. (2018). Enhanced bioreduction-responsive diselenide-based dimeric prodrug nanoparticles for triple negative breast cancer therapy. Theranostics.

[92] He Y., Nie Y., Cheng G., Xie L., Shen Y., Gu Z. (2014). Gene-Delivery Vectors: Viral Mimicking Ternary Polyplexes: A Reduction-Controlled Hierarchical Unpacking Vector for Gene Delivery. Adv. Mater..

[93] Zhang W., Lin W., Zheng X., He S., Xie Z. (2017). Comparing Effects of Redox Sensitivity of Organic Nanoparticles to Photodynamic Activity. Chem. Mater..

[94] Zhang L., Liu Y., Zhang K., Chen Y., Luo X. (2019). Redox-responsive comparison of diselenide micelles with disulfide micelles. Colloid Polym. Sci..

[95] Lin L.S., Song J., Song L., Ke K., Liu Y., Zhou Z., Shen Z., Li J., Yang Z., Tang W. (2018). Simultaneous Fenton-like ion delivery and glutathione depletion by MnO2-based nanoagent to enhance chemodynamic therapy. Angew. Chem..

[96] Zhang M., Jia C., Zhuang J., Hou Y.-Y., He X.-W., Li W.-Y., Bai G., Zhang Y.-K. (2022). GSH-Responsive Drug Delivery System for Active Therapy and Reducing the Side Effects of Bleomycin. ACS Appl. Mater. Interfaces.

[97] Wilson W.R., Hay M.P. (2011). Targeting hypoxia in cancer therapy. Nat. Rev. Cancer.

[98] He H., Zhu R., Sun W., Cai K., Chen Y., Yin L. (2018). Selective cancer treatment via photodynamic sensitization of hypoxia-responsive drug delivery. Nanoscale.

[99] Liu J., Ai X., Cabral H., Liu J., Huang Y., Mi P. (2021). Tumor hypoxia-activated combinatorial nanomedicine triggers systemic antitumor immunity to effectively eradicate advanced breast cancer. Biomaterials.

[100] Perche F., Biswas S., Wang T., Zhu L., Torchilin V. (2014). Hypoxia-targeted siRNA delivery. Angew. Chem..

[101] Yang G., Phua S.Z.F., Lim W.Q., Zhang R., Feng L., Liu G., Wu H., Bindra A.K., Jana D., Liu Z. (2019). A hypoxia-responsive albumin-based nanosystem for deep tumor penetration and excellent therapeutic efficacy. Adv. Mater..

[102] Kulkarni P., Haldar M.K., You S., Choi Y., Mallik S. (2016). Hypoxia-responsive polymersomes for drug delivery to hypoxic pancreatic cancer cells. Biomacromolecules.

[103] Xie Z., Guo W., Guo N., Huangfu M., Liu H., Lin M., Xu W., Chen J., Wang T., Wei Q. (2018). Targeting tumor hypoxia with stimulus-responsive nanocarriers in overcoming drug resistance and monitoring anticancer efficacy. Acta Biomater..

[104] Jiang T., Olson E.S., Nguyen Q.T., Roy M., Jennings P.A., Tsien R.Y. (2004). Tumor imaging by means of proteolytic activation of cell-penetrating peptides. Proc. Natl. Acad. Sci. USA.

[105] Bremer C., Tung C.H., Weissleder R. (2001). In vivo molecular target assessment of matrix metalloproteinase inhibition. Nat. Med..

[106] Cheng Y.-J., Luo G.-F., Zhu J.-Y., Xu X.-D., Zeng X., Cheng D.-B., Li Y.-M., Wu Y., Zhang X.-Z., Zhuo R.-X. (2015). Enzyme-induced and tumor-targeted drug delivery system based on multifunctional mesoporous silica nanoparticles. ACS Appl. Mater. Interfaces.

[107] Gu L., Duan Z., Chen X., Li X., Luo Q., Bhamra A., Pan D., Zhu H., Tian X., Chen R. (2022). A Transformable Amphiphilic and Block Polymer−Dendron Conjugate for Enhanced Tumor Penetration and Retention with Cellular Homeostasis Perturbation via Membrane Flow. Adv. Mater..

[108] Shim M.K., Park J., Yoon H.Y., Lee S., Um W., Kim J.-H., Kang S.-W., Seo J.-W., Hyun S.-W., Park J.H. (2019). Carrier-free nanoparticles of cathepsin B-cleavable peptide-conjugated doxorubicin prodrug for cancer targeting therapy. J. Control. Release.

[109] Cho H., Shim M.K., Moon Y., Song S., Kim J., Choi J., Kim J., Lee Y., Park J.Y., Kim Y. (2022). Tumor-Specific Monomethyl Auristatin E (MMAE) Prodrug Nanoparticles for Safe and Effective Chemotherapy. Pharmaceutics.

[110] Song S.J., Choi J.S. (2022). Enzyme-Responsive Amphiphilic Peptide Nanoparticles for Biocompatible and Efficient Drug Delivery. Pharmaceutics.

[111] Kessenbrock K., Plaks V., Werb Z. (2010). Matrix metalloproteinases: Regulators of the Tumor Microenvironment. Cell.

[112] Quintero-Fabián S., Arreola R., Becerril-Villanueva E., Torres-Romero J.C., Arana-Argáez V., Lara-Riegos J., Ramírez-Camacho M.A., Alvarez-Sánchez M.E. (2019). Role of matrix metalloproteinases in angiogenesis and cancer. Front. Oncol..

[113] Alaseem A., Alhazzani K., Dondapati P., Alobid S., Bishayee A., Rathinavelu A. (2017). Matrix Metalloproteinases: A challenging paradigm of cancer management. Semin. Cancer Biol..

[114] Shahriari M., Zahiri M., Abnous K., Taghdisi S.M., Ramezani M., Alibolandi M. (2019). Enzyme responsive drug delivery systems in cancer treatment. J. Control. Release.

[115] Zhu L., Wang T., Perche F., Taigind A., Torchilin V.P. (2013). Enhanced anticancer activity of nanopreparation containing an MMP2-sensitive PEG-drug conjugate and cell-penetrating moiety. Proc. Natl. Acad. Sci. USA.

[116] Zhou F., Feng B., Yu H., Wang D., Wang T., Ma Y., Wang S., Li Y. (2019). Tumor microenvironment-activatable prodrug vesicles for nanoenabled cancer chemoimmunotherapy combining immunogenic cell death induction and CD47 blockade. Adv. Mater..

[117] Kalafatovic D., Nobis M., Son J., Anderson K.I., Ulijn R.V. (2016). MMP-9 triggered self-assembly of doxorubicin nanofiber depots halts tumor growth. Biomaterials.

[118] Liu Y., Ding X., Li J., Luo Z., Cai K. (2015). Enzyme responsive drug delivery system based on mesoporous silica nanoparticles for tumor therapy in vivo. Nanotechnology.

[119] Hansen A.H., Mouritsen O.G., Arouri A. (2015). Enzymatic action of phospholipase A2 on liposomal drug delivery systems. Int. J. Pharm..

[120] Sun W., Ji W., Hu Q., Yu J., Wang C., Qian C., Hochu G., Gu Z. (2016). Transformable DNA nanocarriers for plasma membrane targeted delivery of cytokine. Biomaterials.

[121] Andresen T.L., Jensen S.S., Jørgensen K. (2005). Advanced strategies in liposomal cancer therapy: Problems and prospects of active and tumor specific drug release. Prog. Lipid Res..

[122] Sharipov M., Tawfik S.M., Gerelkhuu Z., Huy B.T., Lee Y.-I. (2017). Phospholipase A2-responsive phosphate micelle-loaded UCNPs for bioimaging of prostate cancer cells. Sci. Rep..

[123] Ghavami M., Shiraishi T., Nielsen P.E. (2020). Enzyme-triggered release of the antisense octaarginine-pna conjugate from phospholipase A2 sensitive liposomes. ACS Appl. Bio. Mater..

[124] Cao L., Huang C., Zhou D.C., Hu Y., Lih T.M., Savage S.R., Krug K., Clark D.J., Schnaubelt M., Chen L. (2021). Proteogenomic characterization of pancreatic ductal adenocarcinoma. Cell.

[125] Zhang Z.-T., Huang-Fu M.-Y., Xu W.-H., Han M. (2019). Stimulus-responsive nanoscale delivery systems triggered by the enzymes in the tumor microenvironment. Eur. J. Pharm. Biopharm..

[126] Bernardos A., Mondragon L., Aznar E., Marcos M.D., Martinez-Mañez R., Sancenon F., Soto J., Barat J.M., Perez-Paya E., Guillem C. (2010). Enzyme-Responsive Intracellular Controlled Release Using Nanometric Silica Mesoporous Supports Capped with “Saccharides”. Acs Nano.

[127] Clarhaut J., Fraineau S., Guilhot J., Peraudeau E., Tranoy-Opalinski I., Thomas M., Renoux B., Randriamalala E., Bois P., Chatelier A. (2013). A galactosidase-responsive doxorubicin-folate conjugate for selective targeting of acute myelogenous leukemia blasts. Leuk Res..

[128] Rastegari B., Karbalaei-Heidari H.R., Zeinali S., Sheardown H. (2017). The enzyme-sensitive release of prodigiosin grafted β-cyclodextrin and chitosan magnetic nanoparticles as an anticancer drug delivery system: Synthesis, characterization and cytotoxicity studies. Colloids Surf. B Biointerfaces.

[129] Lee J., Jenjob R., Davaa E., Yang S.-G. (2019). NIR-responsive ROS generating core and ROS-triggered 5′-Deoxy-5-fluorocytidine releasing shell structured water-swelling microgel for locoregional combination cancer therapy. J. Control. Release.

[130] Oddone N., Pederzoli F., Duskey J.T., De Benedictis C.A., Grabrucker A.M., Forni F., Vandelli M.A., Ruozi B., Tosi G. (2019). ROS-responsive “smart” polymeric conjugate: Synthesis, characterization and proof-of-concept study. Int. J. Pharm..

[131] Luo C., Sun B., Wang C., Zhang X., Chen Y., Chen Q., Yu H., Zhao H., Sun M., Li Z. (2019). Self-facilitated ROS-responsive nanoassembly of heterotypic dimer for synergistic chemo-photodynamic therapy. J. Control. Release.

[132] Yang B., Wang K., Zhang D., Sun B., Ji B., Wei L., Li Z., Wang M., Zhang X., Zhang H. (2018). Light-activatable dual-source ROS-responsive prodrug nanoplatform for synergistic chemo-photodynamic therapy. Biomater. Sci..

[133] De Gracia Lux C., Joshi-Barr S., Nguyen T., Mahmoud E., Schopf E., Fomina N., Almutairi A. (2012). Biocompatible polymeric nanoparticles degrade and release cargo in response to biologically relevant levels of hydrogen peroxide. J. Am. Chem. Soc..

[134] Sun T., Xu J., Chen T., Tu C., Zhu L., Yan D. (2022). A self-amplified ROS-responsive chemodrug–inhibitor conjugate for multi-drug resistance tumor therapy. Biomater. Sci..

[135] Wang Y., Li C., Du L., Liu Y. (2020). A reactive oxygen species-responsive dendrimer with low cytotoxicity for efficient and targeted gene delivery. Chin. Chem. Lett..

[136] Du Y., He W., Xia Q., Zhou W., Yao C., Li X. (2019). Thioether phosphatidylcholine liposomes: A novel ROS-responsive platform for drug delivery. ACS Appl. Mater. Interfaces.

[137] Heppner G.H., Miller B.E. (1983). Tumor heterogeneity: Biological implications and therapeutic consequences. Cancer Metastasis Rev..

[138] Wicha M.S., Liu S., Dontu G. (2006). Cancer Stem Cells: An Old Idea--A Paradigm Shift. Cancer Res..

[139] Chen M., Liu D., Liu F., Wu Y., Peng X., Song F. (2021). Recent advances of redox-responsive nanoplatforms for tumor theranostics. J. Control. Release.

[140] Sun T., Zhang Y.S., Pang B., Hyun D.C., Yang M., Xia Y. (2021). Engineered nanoparticles for drug delivery in cancer therapy. Nanomater. Neoplasms.

[141] Crommelin D.J.A., Florence A.T. (2013). Towards more effective advanced drug delivery Syst. J. Pharm..

[142] Yang Y., Liu X., Ma W., Xu Q., Chen G., Wang Y., Xiao H., Li N., Liang X.-J., Yu M. (2021). Light-activatable liposomes for repetitive on-demand drug release and immunopotentiation in hypoxic tumor therapy. Biomaterials.

[143] Winkler D.A. (2020). Role of artificial intelligence and machine learning in nanosafety. Small.

[144] Qian Q., Zhu L., Zhu X., Sun M., Yan D. (2019). Drug-polymer hybrid macromolecular engineering: Degradable PEG integrated by platinum (IV) for cancer therapy. Matter.

